# *De novo* p.Arg756Cys mutation of *ATP1A3* causes an atypical form of alternating hemiplegia of childhood with prolonged paralysis and choreoathetosis

**DOI:** 10.1186/s12883-016-0680-6

**Published:** 2016-09-15

**Authors:** Hikaru Kanemasa, Ryoko Fukai, Yasunari Sakai, Michiko Torio, Noriko Miyake, Sooyoung Lee, Hiroaki Ono, Satoshi Akamine, Kei Nishiyama, Masafumi Sanefuji, Yoshito Ishizaki, Hiroyuki Torisu, Hirotomo Saitsu, Naomichi Matsumoto, Toshiro Hara

**Affiliations:** 1 Department of Pediatrics, Graduate School of Medical Sciences, Kyushu University, 3-1-1 Maidashi, Higashi-ku Fukuoka, 812-8582 Japan; 2 Department of Human Genetics, Yokohama City University School of Medicine, Yokohama, Japan; 3 Present address: Fukuoka Children’s Hospital, Fukuoka, Japan; 4 Present address: Section of Pediatrics, Department of Medicine, Fukuoka Dental College, Fukuoka, Japan; 5 Present address: Department of Biochemistry, Hamamatsu University School of Medicine, Hamamatsu, Japan

**Keywords:** Alternating hemiplegia of childhood, Rapid-onset dystonia-Parkinsonism, Areflexia, Optic atrophy and sensorineural hearing loss, Whole-exome sequencing, ATP1A3

## Abstract

**Background:**

Alternating hemiplegia of childhood (AHC) is a rare neurological disorder that manifests recurrent attacks of hemiplegia, oculogyric, and choreoathetotic involuntary movements. *De novo* mutations in *ATP1A3* cause three types of neurological diseases: AHC; rapid-onset dystonia-Parkinsonism (RDP); and cerebellar ataxia, areflexia, pes cavus, optic atrophy, and sensorineural hearing loss (CAPOS) syndromes. It remains to be determined whether or not a rare mutation in *ATP1A3* may cause atypical phenotypes.

**Case presentation:**

A 7-year-old boy presented with recurrent symptoms of generalized paralysis since 1 year and 5 months of age. Hypotonia, dystonia, and choreoathetosis persisted with exacerbation under febrile conditions, but no cerebellar ataxia had ever evolved in 6 years. Whole-exome sequencing (WES) was performed to determine his genetic background, and mutations were validated by the Sanger method. Crude protein extracts were prepared from the cultured cells, and expression of the wild-type or mutant ATP1A3 proteins were analyzed by Western blotting. WES identified a *de nov*o pathogenic mutation in *ATP1A3* (c.2266C > T:p.R756C) for this patient. A literature overview of two reported cases with p.R756C and p.R756H mutations showed both overlapping and distinct phenotypes when compared with those of the present case. The expression of the mutant form (R756C) of ATP1A3 did not differ markedly from that of the wild-type and D801N proteins.

**Conclusions:**

This study confirmed that p.R756C mutation of *ATP1A3* cause atypical forms of AHC-associated disorders. The wide spectra of neurological phenotypes in AHC are linked to as-yet-unknown deficits in the functions of mutant ATP1A3.

**Electronic supplementary material:**

The online version of this article (doi:10.1186/s12883-016-0680-6) contains supplementary material, which is available to authorized users.

## Background

Alternating hemiplegia of childhood (AHC) is a rare neurological disorder characterized by recurrent attacks of hemiplegia and involuntary movements of the body trunk, limbs, and eyes [1]. While the majority of AHC cases are sporadic, the few familial cases suggest that AHC might be inherited as an autosomal dominant pattern [1]. Most patients start suffering from these symptoms in infancy, but the clinical picture varies among cases. Recently, *de novo* mutations in *ATP1A3* have been shown to cause AHC, rapid-onset dystonia-Parkinsonism (RDP), and cerebellar ataxia, areflexia, pes cavus, optic atrophy and sensorineural hearing loss (CAPOS) syndrome [2–13]. Increasing numbers of mutations are being reported, which has helped clarify the phenotypic spectrum of these *ATP1A3*-associated disorders [1, 4, 6, 8]. However, the genotype-phenotype correlations and their pathogenic mechanisms remain elusive [14].

In this report, we present a Japanese boy with AHC who showed mixed phenotypes of AHC with RDP and CAPOS. The clinical signs of this case and a literature overview suggest that an Arg756 mutation of ATP1A3 is linked to the atypical form of AHC with persistent muscle weakness and involuntary movements.

## Case presentation

**Fig. 1:**
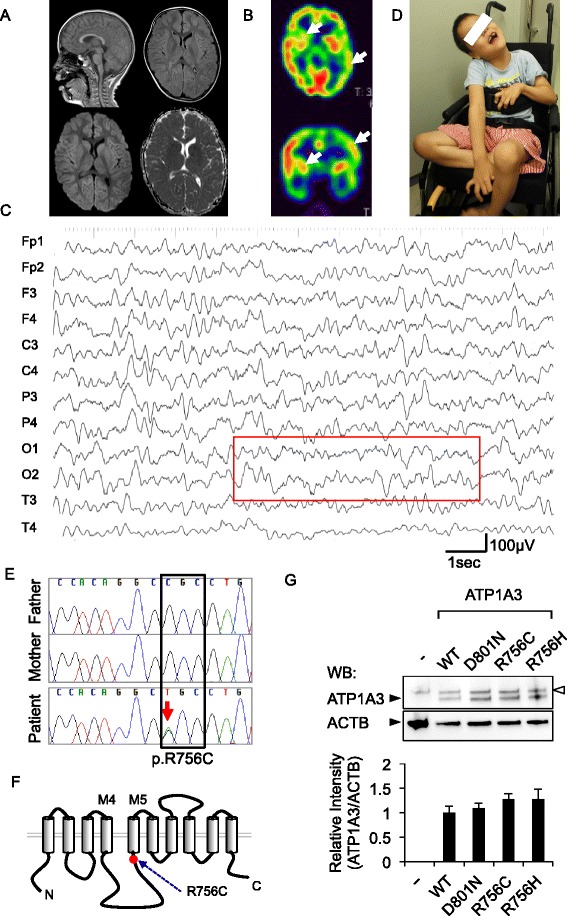
A d*e novo* p.R756C mutation in *ATP1A3* identified in the present case. **a** Brain MRI at the first admission. No parenchymal lesions were detected. Sagittal T1 (upper left), axial fluid-attenuated inversion recovery (upper right), diffusion-weighted image (lower left), and apparent diffusion coefficient map (lower right) images are shown. **b** 99mTc-ECD-SPECT at the 22nd day of admission. The arrows denote the area with decreased perfusion in the left hemisphere and the right striatum with axial (upper) and coronal (lower) images. **c** A representative EEG recording on admission. Note that 8- to 9-Hz alpha rhythms are present over the occipital region (squared), whereas slow-wave bursts and epileptiform discharges are absent in the background. **d** General appearance of the present case at 7 years of age. The photograph shows dystonic posture with prominent choreoathetotic movements of the upper extremities. Written informed consent was obtained from the parents for the use of this image. **e** A *de novo* p.R756C mutation in the present case. Sequence chromatograms for the trio (father, mother, and the patient) are shown. C to T transition (c.2266C > T:p.R756C, red arrow) was present only in the patient and not in either of his parents. **f** The secondary structure of ATP1A3 protein with annotation for the p.R756C mutation. Trans-membrane domains (columns) and cytoplasmic and extracellular loops (black lines) of ATP1A3 are schematically presented. The mutated amino acid residue (red dot) and the two trans-membrane domains (M4 and M5) are annotated for ease of orientation. **g** Representative images of Western blotting. HEK293T cells were transfected either with an empty plasmid (−) or ATP1A3 (WT, D801N and R756C)-encoding plasmids. Specific signals of ATP1A3 (112 kDa) and actin-beta (ACTB, 42 kDa) are annotated with the black arrowheads. Upper bands of ATP1A3 indicate the non-specific signals (white arrowhead). Bar plots represent mean ± SD values for the relative signal intensity of ATP1A3 to that of ACTB (data from three independent experiments)

The present 7-year-old boy was the third offspring of healthy non-consanguineous parents. He was born at 38 weeks of gestational age, weighing 3376 g. No asphyxia or other perinatal events were noted. He was able to control his head at 5 months, sat independently at 8 months, and walked at 14 months of age. When he was infected with influenza at 1 year and 5 months of age, generalized hypotonia and flaccid paralysis rapidly developed within a few hours on the second day of illness. When he was taken by ambulance to the hospital, his respiratory conditions and heart rate were unaltered, but all of his voluntary movements of the eyes, mouth, limbs, and trunk had disappeared. Both eyes remained open, but no verbal or non-verbal responses were made to external stimuli. Bouncing and rapid oculogyric movements in random directions were also prominent signs on admission. Muscle hypotonia was remarkable, while deep tendon reflexes were absent. Hypoglycemia, acidosis, and unbalanced electrolytes were excluded based on the findings of blood gas analyses, blood cell counts, and serum chemistry. Brain magnetic resonance imaging (MRI) showed no parenchymal lesions or atrophy (Fig. 1a). Single-photon emission tomography detected laterality in cerebral blood perfusion on the 22nd day of admission (Fig. 1b). Electroencephalogram (EEG) did not show any high-voltage slow waves, epileptiform discharges, or other signs of encephalopathy (Fig. 1c). From the third week of admission, he began to show voluntary movements in his mouth and hands. Concurrently, choreoathetotic movements in the upper and lower extremities appeared and subsequently continued for over 6 years, until the present. Accordingly, his muscle strength slowly recovered within 2–3 months after the onset, but it never returned to the prior condition. The rapid and random ocular movements were substantially ameliorated within a month and eventually disappeared at 5 years. No signs of cerebellar ataxia were evident throughout the initial admission and thereafter.

He had experienced three episodes of recurrent attacks with flaccid paralysis on febrile illness at 1.9, 3.3, and 5.7 years of age (Additional file 1: Figure S1). In each episode, the involuntary movements disappeared while generalized paralysis persisted for a few weeks. Electroencephalogram (EEG) and MRI studies did not show signs of encephalopathy or neuro-degeneration (data not shown). He had occasional anuresis without signs of paralysis or fever, but his vesicorectal functions were evaluated as normal. At present, he is unable to stand or walk alone, and his daily activity is limited since he uses a wheelchair (Fig. 1d). His verbal skill is severely affected by dysarthria, but he can compose sentences and perform single-digit calculations using a touch-panel display and keyboard. As such, his language perception, social skills, and other cognitive functions were considered minimally affected. He has had no arrhythmic episodes or shown any abnormal features on electrocardiography.

After filtering the polymorphic variations from the WES dataset, we identified two *de novo* mutations in the coding regions of *ATP1A3* (c.2266C > T:p.R756C) and *TOM1L1* (p.Gly4Alafs*16) and one intronic deletion in *C3* (c.1976-22_20TCTdel). All of these mutations were validated by the Sanger method (Fig. 1e and Additional file 1: Figure S2). We considered that the *de novo* missense mutation of *ATP1A3* was likely pathogenic in this case, whereas the effects of the other two events remain to be determined. The variant sequence in *ATP1A3* encodes the protein with an amino acid substitution of Arg756 with Cys. The amino acid residue is located within a conserved sequence across species and was predicted to be deleterious with the Polyphen-2 (http://genetics.bwh.harvard.edu/pph2/), Sift (http://sift.jcvi.org/), and Mutation Taster (http://www.mutationtaster.org/) programs (Additional file 1: Table S5). We further ensured that this mutation was absent in more than 500 healthy individuals. Across the whole protein structure of ATP1A3 (http://www.hprd.org/), the Arg756 residue was located close to the junction of the largest cytoplasmic loop and the fifth trans-membrane domain (Fig. 1f).

The p.R756H mutation, previously reported, causes atypical phenotypes of RPD [12]. Recently, another case carrying a *de novo* p.R756C mutation was shown to have similar clinical features to the present case [4]. To compare the phenotypic spectra of ATP1A3-assocaited disorders, we summarized the clinical features of the reported cases and those of AHC, RDP, and CAPOS (Table 1). The three cases carrying mutations of p.Arg756 to Cys or His all shared the core symptoms of recurrent encephalopathy and acutely developed paralysis followed by prolonged hypotonia, dystonia, and choreoathetosis. We also verified that these cases presented with the mixed phenotypes of AHC, RDP, and CAPOS.

**Table 1:**
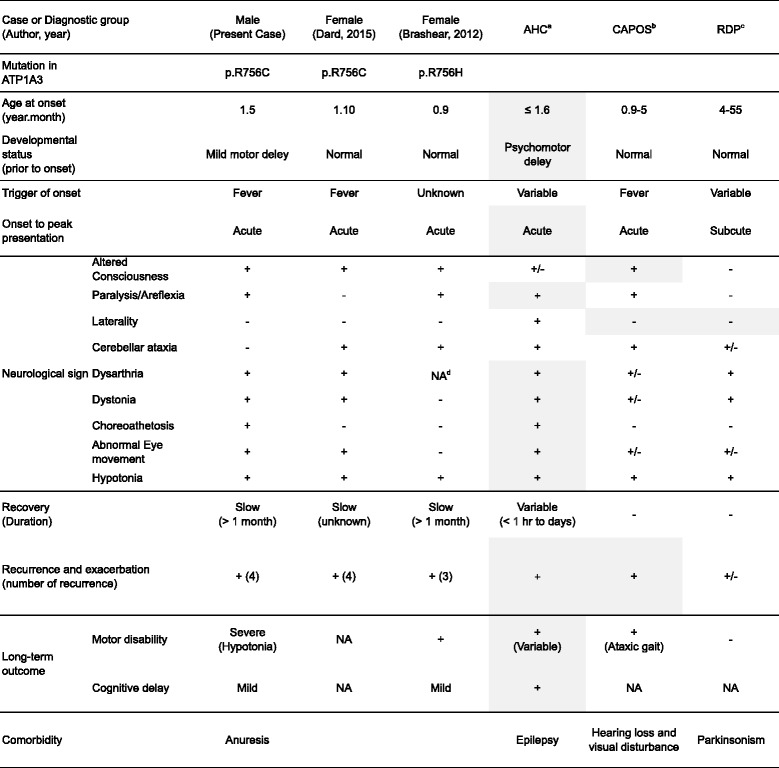
Neurological spectrum of the present and previously reported cases harboring ATP1A3 mutations at Arg756

^a^ *AHC* alternating hemiplegia of childhood

^b^ *RDP* rapid-onset dystonia-parkinsonism

^c^ *CAPOS* cerebellar ataxia, areflexia, pes cavus, optic atrophy and sensorineural hearing loss

*NA* no data available, Overlapping features aer highlighted

Previous studies suggested that RDP-causing mutations were associated with unstable expression of mutant ATP1A3 proteins in cultured cells. We therefore investigated whether or not the p.R756C mutation of ATP1A3 might be expressed at a lower level in HEK293T cells than the wild type and the typical AHC-causing mutant protein (p.D801N). Western blotting showed that the wild type and the two mutant ATP1A3 proteins (p.D801 and p.R756C) were expressed at comparable levels (Fig. 1g).

## Conclusions

The present case showed a unique neurological phenotype that overlapped with those in previous cases carrying the p.R756C or p.R756H mutation of *ATP1A3*. Similarities in their clinical pictures suggested that this mutation is linked to the distinct phenotypes from AHC and other diagnosis of ATP1A3-associated disorders.

In contrast to the previous two cases, the present case did not show cerebellar ataxia. In addition, our case developed involuntary eye movement, one of the key signs of AHC, whereas the other two cases lacked this finding. Although these differences might be subtle, we emphasize in this report that the phenotypic spectrum may vary, even among individuals harboring the same mutation. Previously reported cases with overlapping AHC/RDP or AHC/CAPOS phenotypes suggest that there might be a broad phenotypic spectrum in *ATP1A3*-associated disorders [7, 9, 15, 16]. We propose that p.R756C and other rare mutations in *ATP1A3* may produce an extended phenotype with mixed components of AHC, RDP, and CAPOS (or MARC). Variable phenotypes with the same mutation suggest that the present and previous cases might bear second mutations or inherited causes in their genomes. Regarding non-neurological symptoms, our patient has never presented with arrhythmic episodes or abnormal features on electrocardiography. However, systematic reviews of the cardiac function must be conducted for successful long-term management, as recently reported [17].

When the patient was initially transferred to our department, he was found to have altered consciousness because he did not respond to any neurological examinations or environmental stimuli. However, serial EEG recordings showed regular background activity with no encephalopathy-like slow waves at the acute phase of the recurrent events. These EEG findings strongly suggested that the cortical function was preserved and that acute encephalopathy was unlikely to have been the cause of the atonic events. Given that similar events were observed in the previous two cases, distinguishing severe motor symptoms from encephalopathy may be difficult, due to the systemic, non-focal neurological signs. On the other hand, ECD-SPECT in our case revealed an aberrant local perfusion of cerebral blood flow at the atonic phase. We valued this finding because it implied that the patient had a transiently altered state of consciousness due to thalamic and hippocampal dysfunction.

Using the structural model for the alpha-3 subunit in the Na+/K+ ATPase complex, earlier studies proposed molecular mechanisms through which pathogenic mutations in *ATP1A3* might affect the functions of Na+/K+ ATPase [18, 19]. In the present study, the expression of the p.R756C mutant did not differ markedly from those of wild type and the p.D801N mutant protein in cultured cells, suggesting that this mutation was not critical for protein folding under physiological conditions.

Heinzen et al. demonstrated that introducing RDP-associated mutations in *ATP1A3* resulted in its lower expression in cultured cells [1]. However, our experimental data did not support the hypothesis that R756C mutation might affect the protein stability of ATP1A3. The details regarding the mechanisms that maintain the protein stability remain to be clarified, and no phenotypic modifiers for *ATP1A3* have been identified so far. Given that febrile conditions were associated with recurrent paralysis in our case, the expression and stability of Na+/K+ ATPase protein might fluctuate under inflammatory conditions in the brain. Future translational studies should attempt to identify the key molecular signals that determine the stability of the ATP1A3 protein and clarify their functional roles in vivo.

In summary, this report extended the neurological spectrum of *ATPA1A3*-associated disorders. Further studies will be necessary to clarify they full mutation spectrum of *ATP1A3* and its correlation with the clinical phenotypes of patients. In addition, the accumulation of exome-wide variation data for atypical cases will help improve our understanding of the mechanisms underlying the genotype-phenotype correlations. Linking the genetic findings with the data from biological studies for identifying convergent molecular signals downstream of ATP1A3 will provide new insight into potential targets of therapy in future translational medicine.

## Additional file

Additional file 1: This file contains supplementary methods with one table (**Table S1**), the clinical course of this case (one **supplementary figure 1**), and supplementary data for whole-exome sequencing analysis (**Tables S2-5** and **supplementary figure 2**). (PDF 1180 kb)

## Abbreviations

99mTc-ECD-SPECT: technetium-99 m-ethylcysteinate-dimer single-photon emission computed tomography
AHC: Alternating hemiplegia of childhood
CAPOS: Cerebellar ataxia, areflexia, pes cavus, optic atrophy and sensorineural hearing loss
EEG: Electroencephalogram
MRI: Magnetic resonance imaging
RDP: Rapid-onset dystonia-Parkinsonism
WES: Whole-exome sequencing
Others (gene symbols): *ATPA1A3*: [ATPase Na+/K+ transporting subunit alpha 3; *ACTB* [Actin beta]
