# Supplementary material for: De novo p.Arg756Cys mutation of ATP1A3 causes an atypical form of alternating hemiplegia of childhood with prolonged paralysis and choreoathetosis
Source: BMC Neurol. 2016 Sep 15;16:174. doi: 10.1186/s12883-016-0680-6 (PMC5025569; doi:10.1186/s12883-016-0680-6)
Supplement: Additional file 1: — This file contains supplementary methods with one table (Table S1), the clinical course of this case (one supplementary figure 1), and supplementary data for whole-exome sequencing analysis (Tables S2-5 and supplementary figure 2). (PDF 1180 kb) [file 12883_2016_680_MOESM1_ESM.pdf]

**Additional file**

*De novo* p.Arg756Cys mutation of *ATP1A3* causes an atypical form of alternating hemiplegia of childhood with prolonged paralysis and choreoathetosis

Hikaru Kanemasa, Ryoko Fukai, Yasunari Sakai, Michiko Torio, Noriko Miyake, Hiroaki Ono, Satoshi Akamine, Kei Nishiyama, Masafumi Sanefuji, Yoshito Ishizaki, Hiroyuki Torisu, Hirotomo Saitsu, Naomichi Matsumoto, Toshiro Hara, Shouichi Ohga

This file contains the following items:

- Supplementary methods (Table S1)
- Supplementary figures 1, 2 and their legends
- Supplementary data with tables for the whole-exome sequencing (Tables S2-5)

No additional references are appended to this file.

## Supplementary Methods

Table S1. Summary of the study design and experimental methods

| Item | Protocol and instruments | Vendor |
|---|---|---|
| Ethics | IRB approved (Mitsuo Katano, #461-02) | -[a] |
| Genetics | Kyushu University, #23-53 | - |
| Consent | Written informed consent available | - |
| Whole exome sequencing | Source of DNA: Whole blood (lymphocytes) | - |
| | SureSelect Human All Exon v.4 Kit | Agilent |
| | Data processing: CASAVA v.1.8 | Illumina |
| | Annotation & Mapping: Novoalign | Illumina |
| Site-directed mutagenesis | QuikChange site-directed mutagenesis kit | Agilent |
| Cells & Transfection | HEK293T cells<br>Lipofectamine 3000 | Thermo-Fisher Scientific |
| Antibody | ATP1A3 (sc-374050, 1:1000) | Santa Cruz Biotechnology |
| | ACTB (ab49900, 1:20000) | Abcam |
| Chemiluminescence imager | Alphaimager system | Protein Simple |

[a]-, Not available

Other chemicals and reagents:

After 48 hr of transfection, cells were resuspended in ice-cold hypotonic buffer containing 42 mM KCl, 10mM HEPES pH7.4, 5mM MgCl2, cømplete protease inibitor (Roche) and PhosSTOP phosphatase inhibitor cocktail tablets (Roche), and incubated on ice for 10 minutes. The lysates were centrifuged at 500 xg, 4˚C for 5 minutes to remove nuclei and cell debris. The supernatant was centrifuged at 16,000 xg, 4˚C for 5 min. The pellet was then resuspended in lysis buffer (150 mM NaCl, 20mm TrisHCl pH 7.5, 2mM EDTA, 1% TritonX, cømplete

protease inibitor and PhosSTOP phosphatase inhibitor cocktails) and incubated on ice for 5 minutes. The lysates were centrifuged at 16,000 xg, 4˚C for 10 minutes. After the supernatant was removed, the pellet representing the membrane portion was dissolved in Laemlli sample buffer(BioRad) and mixed well.

The protein samples were subjected to SDS-PAGE (BioRad) without boiling. The protein signal was detected using FluorChem FC2 System (Cell Biosciences) and the protein expression was analysed with FluorChem FC2 software (Cell Biosciences).

## Case report

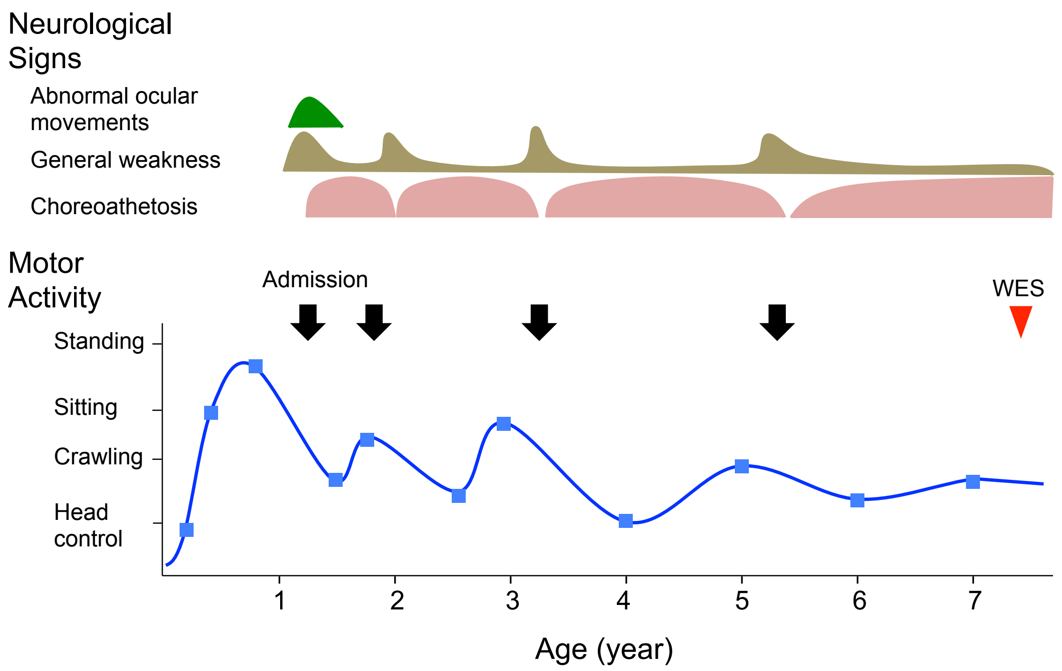


**Supplementary Fig 1**. Clinical course of the present case
This case demonstrated recurrent symptoms of generalized muscle weakness accompanying abnormal ocular movements and involuntary movements. The fluctuated neurological signs are schematically shown at the top. The plots at the bottom represent the semi-quantitative assessment for gross motor activity at the time point (age). Arrows indicate the event of admission. Red triangle denotes the time point of whole-exome sequencing (WES) for this patient and the parents.

**Supplementary data for the whole-exome sequencing**

The mean exon coverage of the WES in this study was 118.94, 85.71, and 78.19 for the case, father, and mother, respectively. The breakdown of the variant calls is shown below (Table S1).

Table S2 Summary of variant calls

| | |
|---|---|
| Total variant calls | 8,571 |
| - segmental duplication | 7,250 |
| - in-house, SNPs | 508 |
| - patient, not include | 260 |
| - variants in father | 127 |
| - variants in mother | 4 |
| - dbSNP137 | 3 |

Thus, the remaining 3 variants were considered as candidates for pathogenic mutations under the *de novo* model. Functional effects of these variants are summarized below (Table S2).

Table S3. Functional effects of three *de novo* variations identified in this case

| Function | Gene | Exonic Function | Amino Acid Change |
|---|---|---|---|
| exonic | *TOM1L1* | frameshift | NM_005486.2:c.11del:p.Gly4Alafs*16 |
| splicing | *C3* | (none) | (NM_000064.3:c.1976-22_20TCTdel) |
| exonic; splicing | *ATP1A3* | non-synonymous SNV | NM_152296:exon17:c.2266C>T:p.R756C |

According to these data, annotated mutations, associated diseases, and the literature information were searched with Human Gene Mutation Database (www.hgmd.cf.ac.uk). The results are summarized in Table S3. As shown in this

table, only ATP1A3 was linked to the Mendelian disorder that has been reported in the literature.

Table S4. Annotated mutations in HGMD

| Gene | HGMD_GENE (2013.03) |
|---|---|
| *TOM1L1* | Not available |
| *C3* | Temporal lobe epilepsy & febrile seizures, protection, assoc with:C3:Complement component 3:(CA)n in promoter:DFP:Jamali:PLoS One:5:e12740:2010: 20862287:PRI:CE106036:E |
| *ATP1A3* | Dystonia-parkinsonism, rapid-onset:ATP1A3:ATPase, Na+/K+ transporting, alpha 3 polypeptide:ins 3 bp codon 1013:DM:Blanco-Arias:Hum Mol Genet:18:2370:2009: 19351654:PRI:CI093547:I |

These variants were validated with Sanger sequencing. The sequence chromatograms for TOM1L1 and C3 are shown in supplementary figure 2. Damaging scores of the missense mutation in *ATP1A3* were calculated with open resources and are summarized in Table S5.

Table S5. Damaging scores with p.R756C mutation of ATP1A3

| Methods | Accession/Protein ID | Score | Result |
|---|---|---|---|
| Polyphen-2 | P13637 | 1.0 | Probably Damaging |
| SIFT | ENSP00000302397 | 0 | Damaging |
| Mutation Taster | ATP1A3 | 180 | Disease Causing |

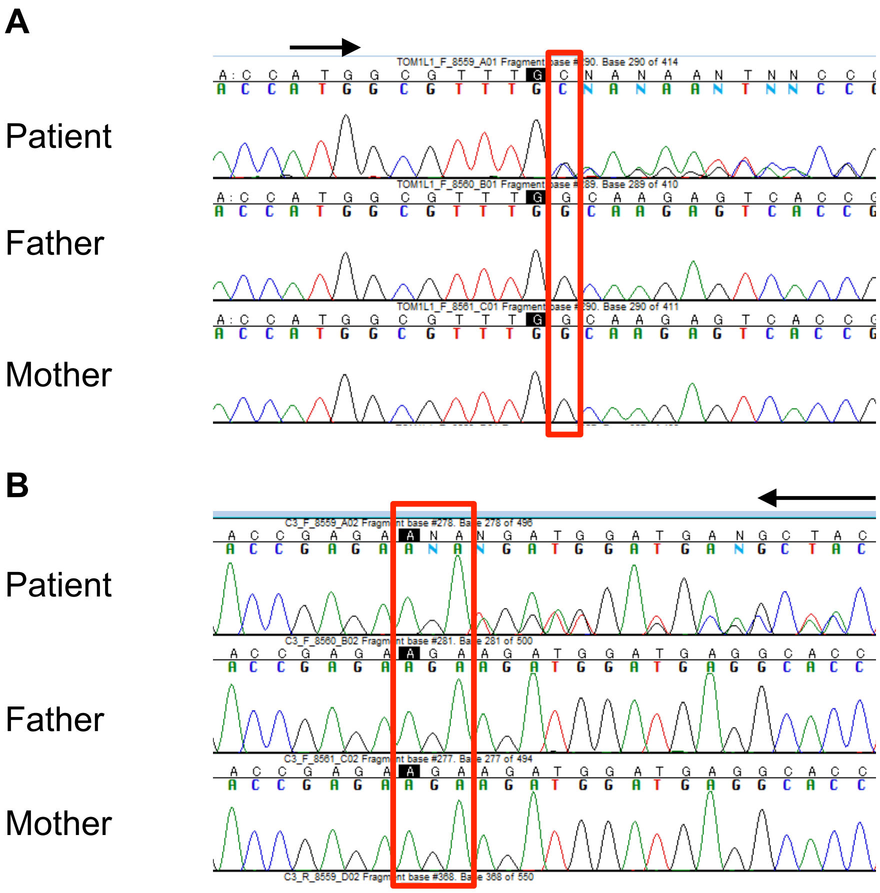


**Supplementary figure 2.** Validation of *de novo* mutations by Sanger sequence Sequence chromatograms for TOM1L1: NM_005486.2:c.11del:p.Gly4Alafs*16 (A) and C3: NM_000064.3:c.1976-22_20TCTdel (B) are shown. Red rectangles highlight the position of *de novo* events. Arrows indicate the direction of coding sequences.
